# Optimizing the Rigid or Compliant Behavior of a Novel Parallel-Actuated Architecture for Exoskeleton Robot Applications

**DOI:** 10.3389/frobt.2021.596958

**Published:** 2021-02-23

**Authors:** Justin Hunt, Hyunglae Lee

**Affiliations:** School for Engineering of Matter, Transport and Energy, Arizona State University, Tempe, AZ, United States

**Keywords:** Parallel actuation, parallel mechanism, exoskeleton robotics, shoulder exoskeleton, stiffness optimization, compliant optimization

## Abstract

The purpose of this work is to optimize the rigid or compliant behavior of a new type of parallel-actuated robot architecture developed for exoskeleton robot applications. This is done in an effort to provide those that utilize the architecture with the means to maximize, minimize, or simply adjust its stiffness property so as to optimize it for particular tasks, such as augmented lifting or impact absorption. This research even provides the means to produce non-homogeneous stiffness properties for applications that may require non-homogeneous dynamic behavior. In this work, the new architecture is demonstrated in the form of a shoulder exoskeleton. An analytical stiffness model for the shoulder exoskeleton is created and validated experimentally. The model is then used, along with a method of bounded nonlinear multi-objective optimization to configure the parallel substructures for desired rigidity, compliance or nonhomogeneous stiffness behavior. The stiffness model and its optimization can be applied beyond the shoulder to any embodiment of the new parallel architecture, including hip, wrist and ankle robot applications. In order to exemplify this, we present the rigidity optimization for a theoretical hip exoskeleton.

## 1 Introduction

In the field of exoskeleton robotics, parallel actuation can offer many advantages over more commonly used serial actuation. Despite having complex kinematics and a typically small workspace, parallel actuation has numerous useful properties including low end-effector inertia, high acceleration, high position accuracy, and the potential for high stiffness (Li and Bone, 2001; Merlet, 2012; Taghirad, 2013). Furthermore, certain types of parallel architectures, such as the 3-SPS (spherical-prismatic-spherical) (Alici and Shirinzadeh, 2004), 3-RRR (revolute-revolute-revolute) (Wu et al., 2011) and 3-UPU (universal-prismatic-universal) (Di Gregorio, 2003), can operate without occupying the center of rotation, which is particularly useful when interfacing with multiple degrees-of-freedom (DoF) biological joints such as the ankle, hip, shoulder and wrist.

Parallel actuation has been utilized for a number of exoskeleton applications. These include devices for the wrist, ankle, hip and shoulder. The wrist exoskeleton RiceWrist (Gupta et al., 2008), uses a 3-RPS (revolute-prismatic-spherical) architecture with an additional serial revolute joint to generate 4-DoF. These DoF include the rotation of the forearm, wrist height and 2-DoF in rotation of the end-effector platform. Since the introduction of the RiceWrist, several other exoskeleton research prototypes have adopted the 3-RPS architecture (Fan and Yin, 2009; Nurahmi et al., 2017). The ankle exoskeleton Anklebot (Roy et al., 2009) uses a 2-SPS-1S (spherical-prismatic-spherical, spherical) manipulator in conjunction with the ankle joint to achieve semi-spherical motion. The shoulder exoskeleton BONES (Klein et al., 2010) uses a RRPS (revolute-revolute-prismatic-spherical) manipulator to achieve spherical motion. Because all of these architectures, along with the previously mentioned 3-SPS, 3-RRR and 3-UPU, generate spherical motion through parallel actuation, they can further be categorized as spherical parallel manipulators.

Spherical parallel manipulators (SPMs) are the most popular choice for exoskeleton applications, primarily because they offer a greater workspace than parallel architectures with a high degree of actuation, like the Stewart-Gough Platform (Stewart, 1965). This is a result of SPMs typically having two to three actuated substructures instead of the four, five or six of typical of higher DoF parallel manipulators. This means that SPMs have less mechanical interference between substructures. However, fewer active DoF also means that SPMs typically have lower stiffness performance than higher active DoF parallel manipulators (Gosselin and Angeles, 1989; Jiang and Gosselin, 2009; Walter et al., 2009). This can be problematic, particularly for augmentative exoskeleton systems that require high rigidity.

In order to improve the workspace/stiffness tradeoff of SPMs, the authors introduced a new type of SPM architecture (Hunt et al., 2017). The architecture utilizes a new design method that the authors refer to as modular motion coupling (MMC). The method involves coupling multiple DoF of each actuated substructure in order to maintain a high level of actuation while still maintaining a relatively low number of substructures. The authors developed a shoulder exoskeleton prototype that utilized this new architecture and performed a stiffness analysis on it (Hunt et al., 2018). Many approaches to analyzing the stiffness of parallel manipulators have been proposed over the years. One popular method utilizes the Jacobian matrix to calculate the stiffness matrix (Gosselin, 1990). While this method provides a reasonable approximation of stiffness, it does not take into account linkage flexibility, which is critical for an accurate end-effector stiffness estimate. Another method utilizes strain energy to develop a model of stiffness (Yan et al., 2016). While promising, this strain energy method is quite new and therefore less proven than other solutions. Additional methods include a lumped parameter approach (Pashkevich et al., 2009) and a more traditional FEA approach (El-Khasawneh and Ferreira, 1999). After considering each of these, the authors opted for a different method that utilized matrix structural analysis techniques that have been used extensively in civil engineering and have been proven to provide accurate estimates of end-effector stiffness for parallel manipulators with both passive and active DoF and flexible linkages (Deblaise et al., 2006). The results of the stiffness analysis identified some non-homogeneous stiffness behavior for certain end-effector orientations of the MMC design. This was determined to be a result of each substructure not having an actuated roll DoF. In addition, the MMC architecture was non-backdrivable, which limited its number of practical applications. Having identified these limitations, the authors developed a second-generation SPM that resolved these issues (Hunt and Lee, 2018; Hunt and Lee, 2019).

The second-generation SPM developed by the authors utilized a system of 4-bar (4B) mechanisms to rotate a mobile platform about a center point. The advantage of this new 4B-SPM design is that the 4-bar system achieves similar arc motion to the previous design while utilizing a far more simplistic construction and maintaining back-drivability. Furthermore, the 4B-SPM utilizes three additional motors to actuate the roll DoF of each substructure, eliminating the primary issues of the MMC design.

An additional property of the 4B-SPM architecture is flexibility of actuator placement. The three substructures that comprise the device can be placed in any position about a center point. Placement is critical, as the stiffness of the 4B-SPM will be highly dependent on the configuration chosen. Therefore, a stiffness model with substructure placement as an input and end effector stiffness as an output would be useful for achieving desired dynamic behavior. Several examples of this include:

1. Maximizing stiffness for applications such as lifting or crush protection.
2. Maximizing compliance for applications requiring a high degree of unpredictable human-robot interaction or collision protection.
3. Designing custom non-homogeneous stiffness ellipsoids for applications that may require non-homogeneous dynamic behavior.

With a stiffness model, the 4B-SPM could have widespread application for exoskeleton devices, as it has been shown to 1) interface well the shoulder, hip, wrist and ankle, 2) not require any complex mechanical components, 3) have very flexible actuator placement, and 4) not require the human joint for a singular kinematic solution (Hunt and Lee, 2018). For this reason, a 4B-SPM stiffness model is developed and presented in this work. It should be noted that, as previously mentioned, the authors have developed stiffness models for past parallel architectures. However, the ability of the 4B-SPM to interface well with different biological joints, along with its economic design, makes it a major improvement over past parallel architectures development by the authors. Therefore, a separate stiffness analysis of this architecture is justified as it would offer other researchers and members of the robotics community a complete and flexible parallel actuated solution that could be customized to fit many different exoskeleton design requirements.

The rest of this paper presents the steps taken to optimize the rigid or compliant behavior of the 4B-SPM for a given workspace. The sections are organized as follows: Section 2 includes 1) a brief overview of the of the 4B-SPM architecture, 2) the model used to characterize stiffness, 3) the experimental setup to validate the stiffness model, and 4) the optimization techniques used to maximize the rigid, compliant or nonhomogeneous stiffness behavior of the 4B-SPM. Section 3 details 1) the results of the stiffness model validation experiment, 2) the optimal actuator placement for maximum rigid, compliant or nonhomogeneous stiffness behavior of a 4B-SPM shoulder exoskeleton embodiment, and 3) the maximum rigid stiffness of a 4B-SPM hip exoskeleton embodiment. Finally, Section 4 concludes the paper with a discussion and summary of the contribution.

## 2 Methods

### 4B-Spherical Parallel Manipulator Design Overview

**FIGURE 1:**
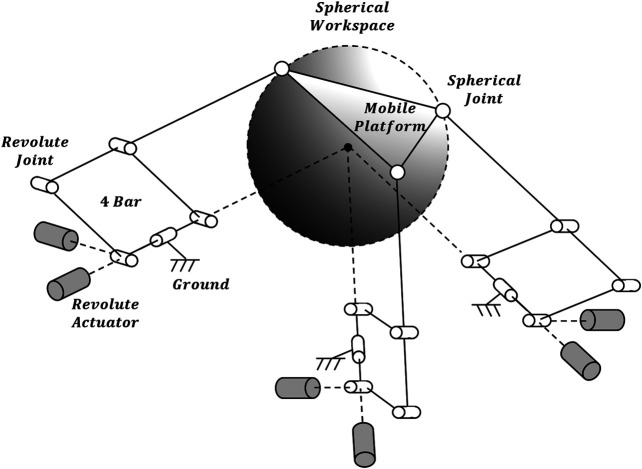
4-Bar Spherical Parallel Manipulator (4B-SPM) architecture. The 4B-SPM uses three parallelogram 4-bar substructures. Each substructure has two actuated DoF: pitch and roll. The roll DoF axis of each substructure intersects with the others at a singular point which represents the virtual center of a spherical workspace. The top linkage in each 4-bar substructure is extended to reach a mobile platform that moves tangential to the spherical workspace. Each top linkage is coupled to the mobile platform using a spherical joint (Hunt et al., 2017).

**FIGURE 2:**
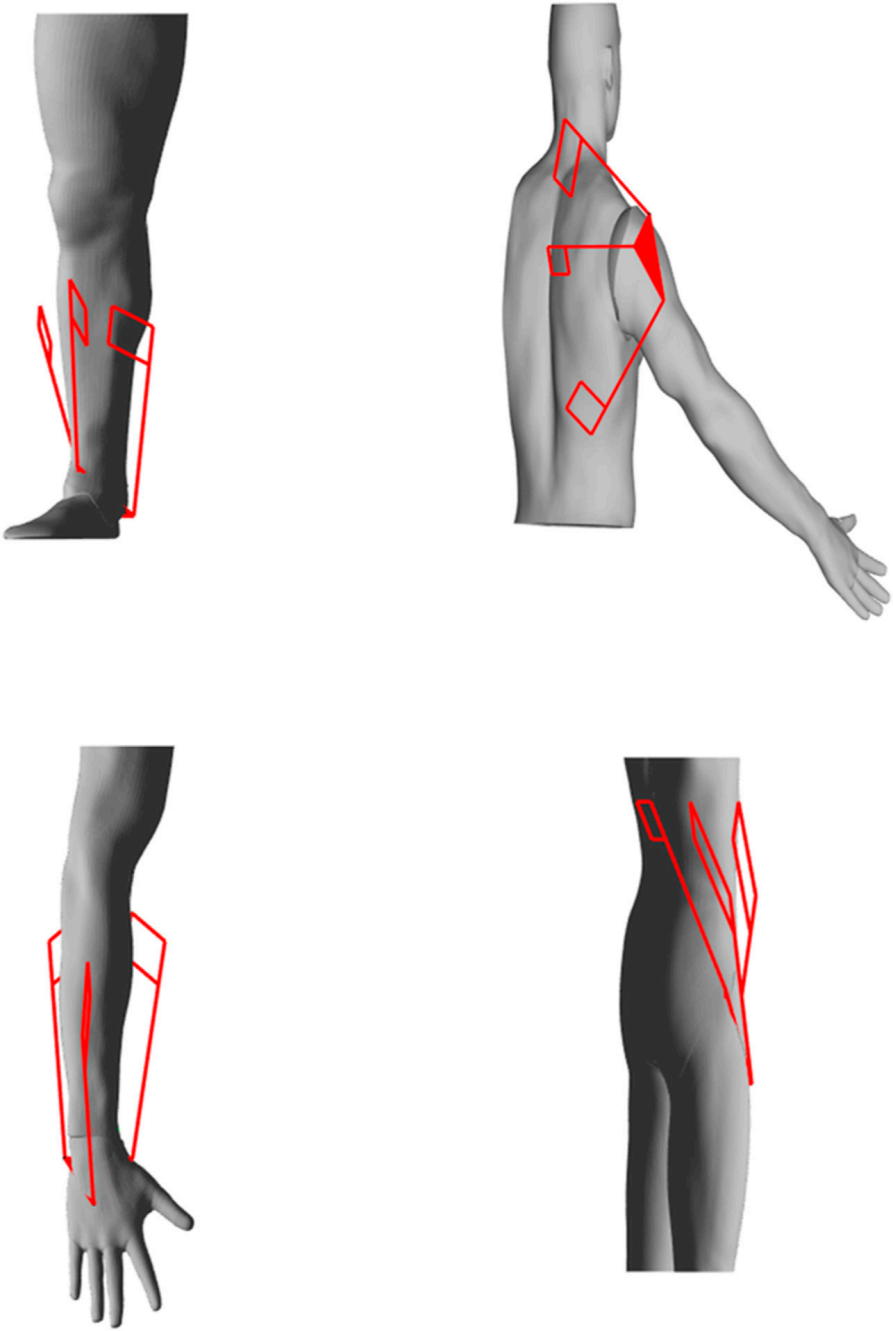
Four embodiments of the 4B-SPM architecture for which the authors have solved the kinematics for include: ankle, shoulder, wrist and hip exoskeletons (Hunt et al., 2017).

**FIGURE 3:**
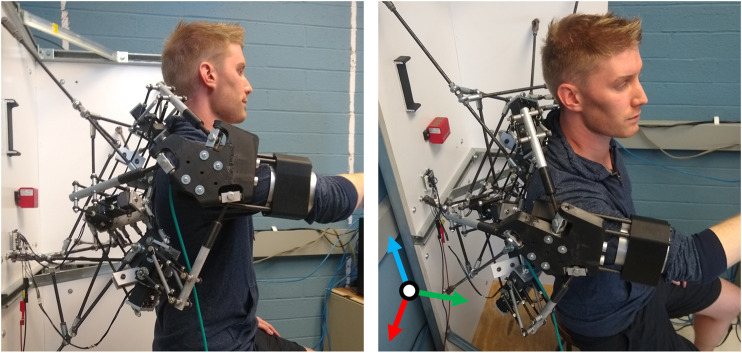
4B-SPM shoulder exoskeleton prototype mounted to a stationary platform with a human subject in the seated position. The subject is coupled to the device through the use of an upper arm cuff. To maintain good contact between the subject and device, a blood pressure cuff is used at the contact point. The pitch, roll and yaw axes are represented by the orthogonal red, green and blue axes, respectively.

The previously developed 4B-SPM architecture is presented in Figure 1 (Hunt and Lee, 2018). The 4B-SPM uses three parallelogram 4-bar substructures. Each substructure has two actuated DoF: pitch and roll. The roll DoF axis of each substructure intersects with the others at a singular point which represents the virtual center of a spherical workspace. The top linkage in each 4-bar substructure is extended to reach a mobile platform that moves tangential to the spherical workspace. Each top linkage is coupled to the mobile platform using a spherical joint. Shown in Figure 2 are four different embodiments of the 4B-SPM architecture that the authors have developed forward and inverse kinematic models for (Hunt and Lee, 2018). In preparation for the dynamic analysis performed in this work, the authors developed a shoulder exoskeleton prototype of the 4B-SPM architecture (Hunt and Lee, 2019). This prototype is shown in Figure 3. A video of the shoulder exoskeleton is included as an attachment to this work.

### 4B-Spherical Parallel Manipulator Stiffness Model

For the purpose of determining end effector stiffness of the 4B-SPM for different substructure configurations, an analytical model was created. The model is based off of a matrix structural analysis method commonly used for calculating stiffness of complex truss networks typically found in bridges. The concept of applying this method to parallel manipulators was first introduced by Dominique Debase. For brevity, the reader will be referred back to Debase’s prior work for some of the more derivative or expansive steps required in the development of this model. With the model, it is possible to generate the end effector rotational stiffness ellipsoids that will govern how the 4B-SPM responds to externally applied torques. ${\text{θ}}_{\text{k}}$θkθk.

**FIGURE 4:**
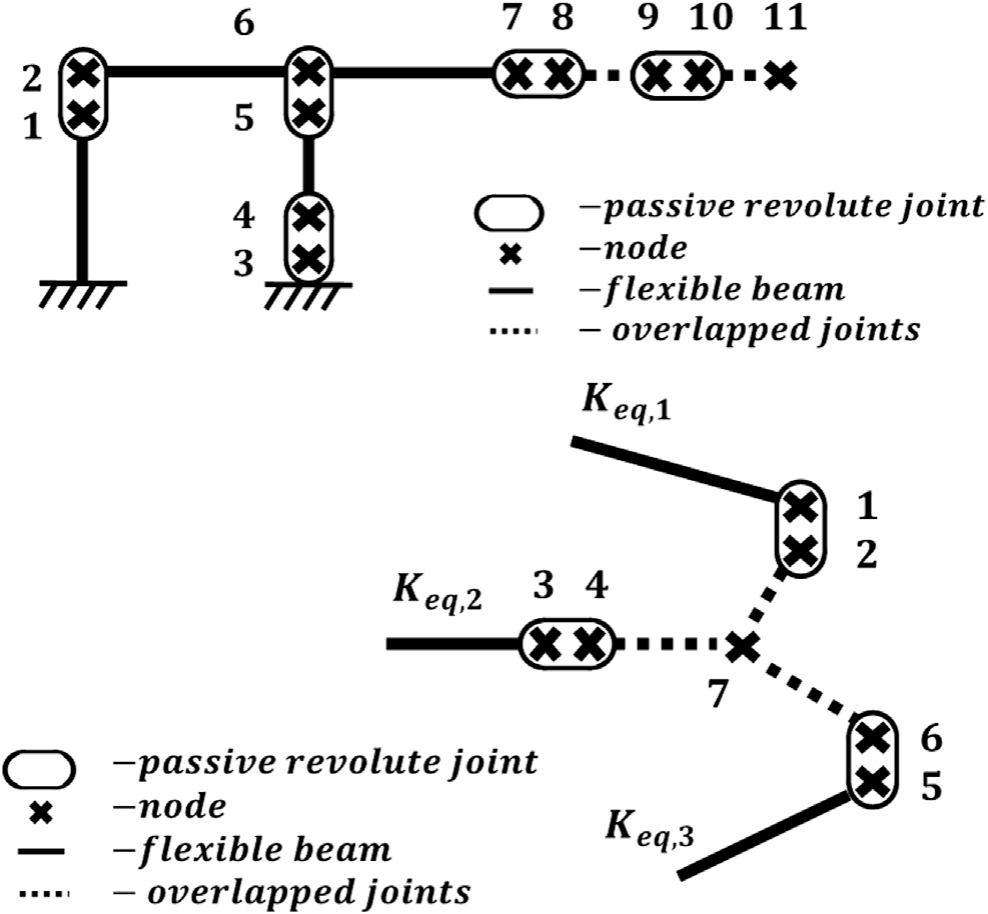
**(Top)** 4-bar substructure equivalent nodal diagram **(Bottom)** shoulder plate end effector equivalent nodal diagram.

To start, each actuated substructure k (k=1, 2, 3) is represented by a nodal system that corresponds to characteristic points. Shown in Figure 4 are the node locations for each substructure. It should be noted that a simplification has been made to the nodal diagram with regards to the 4-bar mechanism. In the prototype shown in Figure 3, there are actually four parallel vertical bars connecting the top and bottom linkage of the 4-bar mechanism, whereas the nodal diagram shown in Figure 4 reduces this down to two. This is done to simplify the analysis and is justified by the fact that only one of the four parallel vertical bars is actually connected to the servo motor and therefore grounded, similar to Figure 4. Thus, pitch and roll stiffness of the substructure will not be affected by this simplification. The yaw may be slightly affected, although it is not considered to be of the same contributing magnitude to the overall stiffness model as pitch and roll. Nevertheless, to mitigate this error, the authors make an adjustment to the geometric properties of the two vertical bars within the model to more accurately reflect the actual prototype.

The nodes shown in Figure 4 are coupled by either a flexible beam or passive revolute joint. Each beam n is fixed at its ends by one or two nodes, depending on if the beam is considered rigidly fixed at one end. Therefore, each beam is represented by either a 6 × 6 or the 12 × 12 beam stiffness matrix $K_{n,k}$ as defined in Euler–Bernoulli beam theory. Each of these beam stiffness matrices must be oriented through multiplication of matrix $P_{n,k}$ comprised of rotational submatrices $R_{n,k}$ along its diagonal. The rotated beam stiffness matrix $K{'}_{n,k}$ can be expressed as:

$$K{'}_{n,k}={P_{n,k}}^{-1}K_{n,k}P_{n,k}$$ (1)

Where rotation matrix $P_{n,k}$ can be determined by:

$$P_{n,k}=\left[\begin{array}{ccc} R_{n,k} & 0 & \cdots \\ 0 & R_{n,k} & \cdots \\ \vdots & \vdots & \ddots \end{array}\right]$$

The n number of rotated beam stiffness matrices $K{'}_{n,k}$ can then be assemble into a singular substructure stiffness matrix $K_{T,k}$. This assembly can be done using recognized stiffness matrix assembly methods.

The substructure stiffness matrix $K_{T,k}$ represents substructure stiffness before the addition of passive joints shown in Figure 4. Each passive joint will be defined by a kinematic relationship matrix $A_{n,k}$, which can be expressed as:

$$A_{n,k}=\left[\begin{array}{cc} I_{3x3} & 0_{3x3}\\ 0_{2x3} & r_{n,k} \end{array}\right]$$ (2)

Where $r_{n,k}$ is comprised of the rotation matrix vectors orthogonal to the rotation axis unit vector of the passive joint. One of these rotation matrix vectors should also be parallel to the adjacent beam. The $A_{n,k}$ matrices can then be assembled into a singular substructure kinematic matrix$\;A_{T,k}$, similar to$\;K_{T,k}$. The kinematically adjusted substructure stiffness matrix, with the inclusion of passive joints, is derived using the minimum total potential energy principle (Deblaise et al., 2006). It can be expressed as:

$$K_{G,k}=\;\left[\begin{array}{cc} K_{T,k} & {A_{T,k}}^{T}\;\\ A_{T,k} & 0 \end{array}\right]$$ (3)

At this point, it is necessary to permutated $K_{G,k}$ in order to move the last node submatrix to the end of the $K_{G,k}$ so that it can be redefined as the endpoint substructure stiffness matrix $K_{eq,k}$ *.*

**FIGURE 5:**
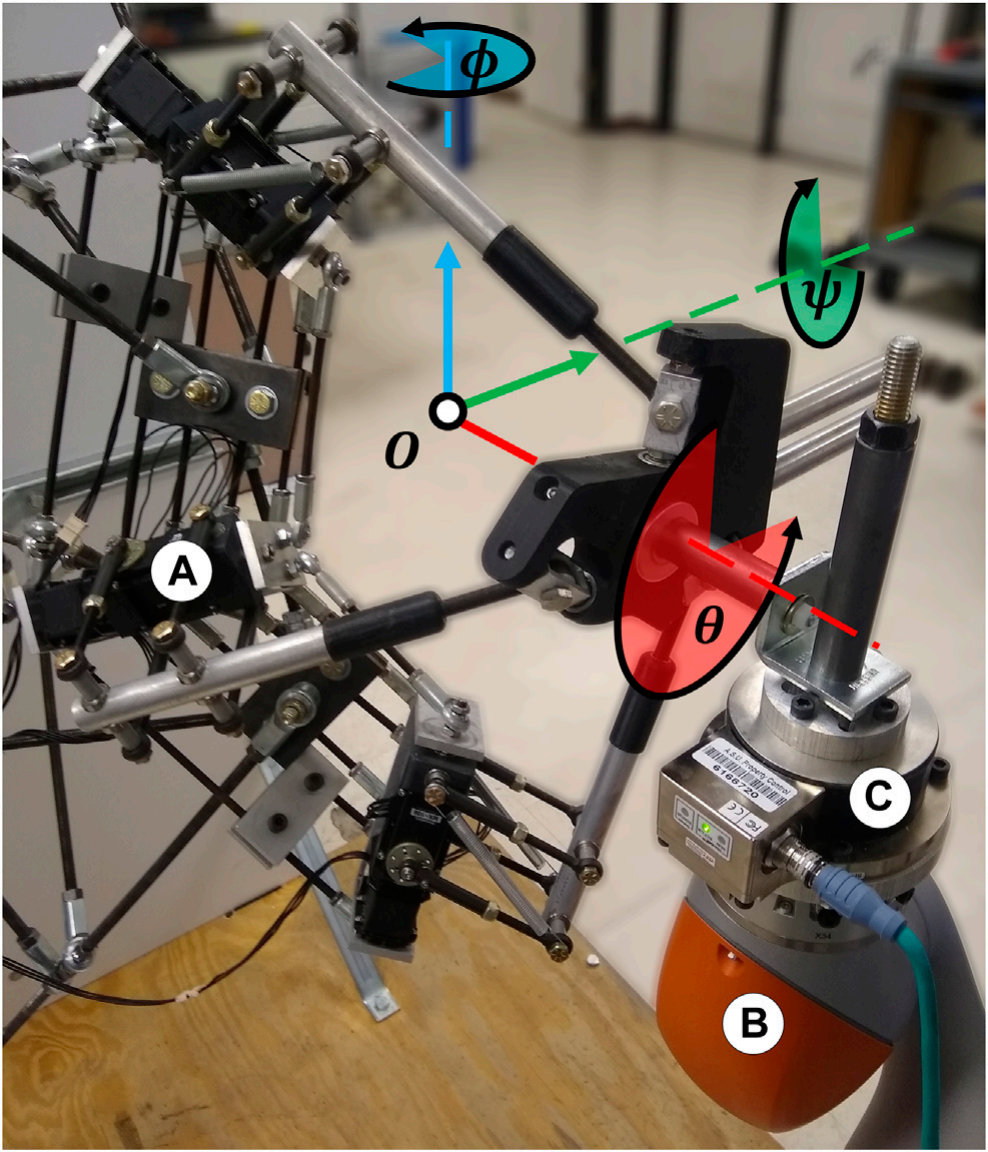
**(A)** Shown at top is the generalized maximum stiffness configuration for the 4B-SPM shoulder exoskeleton substructures along with point clouds of the best solutions found throughout the workspace. Shown at bottom are projections of the generalized maximum stiffness ellipsoid. **(B)** Shown at top is the generalized minimum stiffness configuration for the 4B-SPM substructures along with point clouds of the best solutions found throughout the workspace. Shown at bottom are projections of the generalized minimum stiffness ellipsoid. **(C)** Shown at top is the generalized maximum desired nonhomogeneous stiffness configuration for the 4B-SPM substructures along with point clouds of the best solutions found throughout the workspace. Shown at bottom are projections of the generalized maximum nonhomogeneous stiffness ellipsoid. For all three figures, the origin of each frame is at the center-of-rotation of the human shoulder.

In order to determine the global stiffness of the 4B-SPM architecture, the substructure end point stiffness matrices $K_{eq,k=1,2,3\;}$ must be assembled to the end effector node 7 shown in Figure 5. The shoulder plate that connects $K_{eq,k=1,2,3\;}\;$is considered rigid and therefore cannot be modeled using Euler–Bernoulli beam theory. Instead, it will be modeled as series of rigid beams with infinite stiffness. This rigid beam model will be defined by the kinematic relationship matrix$\;B_{n}$, which can be expressed as:

$$B_{n}=\left[\begin{array}{cc} 0_{3x3} & I_{3x3}\\ I_{3x3} & {\hat{L}}_{W_{n}} \end{array}\right]$$ (4)

Where ${\hat{L}}_{W_{n}}$ is the symmetric skew matrix defined by the rigid beam direction vector$\;W_{n}={\left[L_{x}\;L_{y}\;L_{z}\right]}_{n}{}^{T}$. With the kinematic relationship matrix $B_{n}\;$defined, the kinematic relation matrix $A_{T}$ of the shoulder plate can be constructed in a similar manner to $A_{T,k}$. The shoulder plate stiffness matrix $K_{T}$. Can also be constructed in a similar to $K_{T,k}$. The kinematically adjusted shoulder plate stiffness matrix, with the inclusion of passive joints and rigid beams, is once again derived using the minimum total potential energy principle:

$$K_{eq,T}=\;\left[\begin{array}{cc} K_{T} & A_{T}T\;\\ A_{T} & 0 \end{array}\right]$$ (5)

Similar to $K_{G,k}$, it is necessary to permutated $K_{eq,T}$ in order to move the last node submatrix to the end so that it can be redefined as the 6 × 6 end-effector stiffness matrix $K_{ee}$, which represents the stiffness at node 7 in Figure 4.

The end-effector stiffness matrix $K_{ee}$ can be visualized by plotting its translational and rotational stiffness ellipsoids. As defined in the work of Mussa-Ivaldi, these ellipsoids are created by first decomposing $K_{ee}$ into its symmetric $K_{s}$ and an antisymmetric $K_{a}$ component*.* Assume that $K_{ee}\;$is defined by the following four submatrices:

$$K_{ee}\;=\;\left[\begin{array}{cc} K_{xx} & K_{xy}\;\\ K_{yx} & K_{yy} \end{array}\right]$$ (6)

Then $K_{s}$ and $K_{a}$ can be written as:

$$K_{s}=\;\left[\begin{array}{cc} K_{xx} & \frac{K_{xy}+K_{yx}}{2}\;\\ \frac{K_{yx}+K_{xy}}{2} & K_{yy} \end{array}\right]$$ (7)

$$K_{a}=\;\left[\begin{array}{cc} 0 & \frac{K_{xy}-K_{yx}}{2}\;\\ \frac{K_{yx}-K_{xy}}{2} & 0 \end{array}\right]$$ (8)

where $K_{ee}=K_{s}+K_{a}$. The first three eigenvalues and eigenvectors of $K_{s}$ represent the direction and magnitude of the three pairwise perpendicular axes of symmetry for the translational stiffness matrices. The last three correspond to the perpendicular axes of symmetry of the rotational stiffness ellipsoid.

### Stiffness Model Testing

**FIGURE 6:**
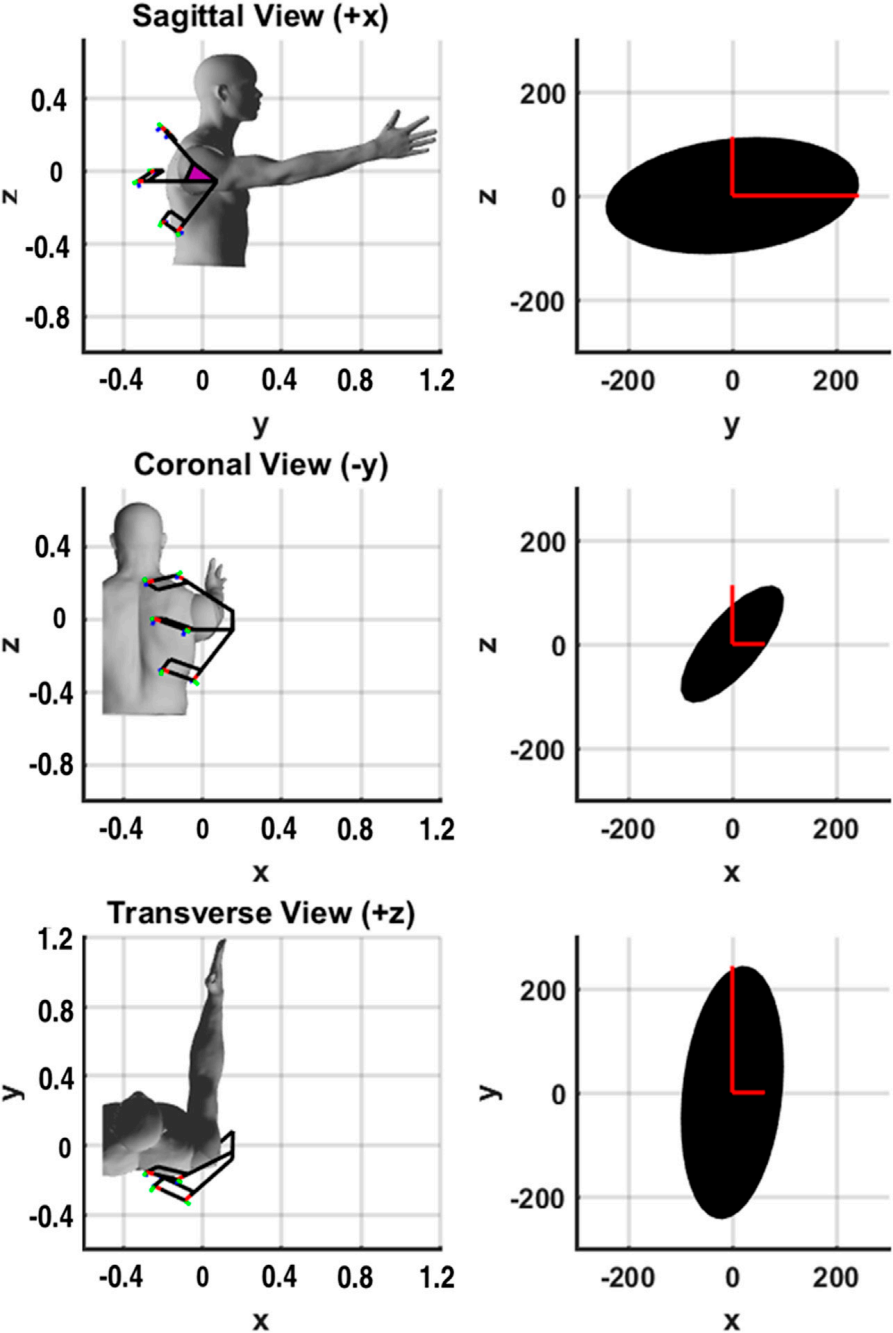
Experimental setup for evaluating the 4B-SPM prototype stiffness oriented at 90° flexion. **(A)** 4B-SPM Shoulder exoskeleton **(B)** 7-DoF robotic arm (LBR iiwa R820, KUKA, Germany) **(C)** 6-axis load cell (Delta IP65, ATI, NC). The shoulder exoskeleton was mechanically coupled to the load cell, which was in turn coupled to the 7-DoF robotic arm. The roll, pitch and yaw angles of the shoulder exoskeleton about its center-of-rotation O are represented ψ, *θ* and ϕ, respectively.

An experiment was performed to test the validity of the stiffness model through a comparison of the theoretical 4B-SPM stiffness to that of the prototype. The shoulder exoskeleton was oriented at 90° flexion and coupled to one end of a 6-axis force/torque sensor (Delta IP65, ATI, NC). To provide an accurate displacement of the load cell, a 7-DoF research robotic arm (LBR iiwa R820, KUKA, Germany) was connected to the other end of the sensor. This robot was chosen for its ability to perform these sensitive experiments. In addition to a rated payload that exceeds to forces exerted during these tests, the device has highly repeatable position control (±0.015 mm), which is necessary for accurate stiffness estimates (KUKA Robot Group, 2015). The 7-DoF robotic arm was in turn bolted to a steel structural support column. The experimental setup is shown in Figure 6.

The roll (*ψ*), pitch (*θ*) and yaw (*ϕ*) angles of the shoulder exoskeleton were perturbed ±3° by the 7-DoF robotic arm. A sinusoidal perturbation profile commanded over 3,000 ms was used. The corresponding forces were recorded by the 6-axis load cell at 1 kHz. All the collected measurements were filtered using a zero-phase 2nd order Butterworth filter with a 20 Hz cutoff frequency. With measurements of corresponding displacement Δθ and force F, it is possible to calculate the stiffness k of the prototype using $F_{\text{θ}}=k\Delta \text{θ}.\;$Peak displacement and the corresponding force were used for calculating stiffness. It should be noted that the theoretical stiffness model is a function of the kinematic relationship matrix$\;A_{T}\;\;$and stiffness matrix $K_{T}$. These matrices are sensitive to change, so if it were incorrect, then significant differences from the theoretical stiffness model and prototype would be expected.

For the simulation, all flexible beams were modeled as 1,045 carbon steel, except for the top linkage that was modeled as 2024 aluminum. This is representative of the materials used for the prototype. All critical dimensions used in the simulation match those of the prototype. The only exception to this was the flexible beam connecting nodes 4 and 5 of the 4-bar mechanism shown in Figure 4. For the reasons mentioned in the beginning of this Section, the cross-sectional area of this beam was doubled to more accurately reflect the duel beam design used in the prototype.

### Stiffness Optimization

In order to maximize overall rigidity, compliance, or nonhomogeneous stiffness behavior for a given workspace, the placement of each substructure (i.e., XYZ mounting locations of each actuator) needs to be optimized. There are a couple of parameters applied to this optimization. First, solutions for each substructure location must be bounded to a practical region were mechanical interference between robot-robot and human-robot cannot occur. After considering the geometry of the human model shown in Figure 4 and the approximate workspace of the human shoulder, the regions [$-0.3<x_{t}<0.1,$ $-0.4<y_{t}<0,\;\;$ $0<z_{t}<0.3$] m [$-0.3<x_{m}<0.1,$ $-0.4<y_{m}<0,\;$ $-0.3<z_{m}<0.1$] m, and [$-0.4<x_{b}<0.1,$ $-0.4<y_{b}<0,\;$ $-0.4<z_{b}<-0.2$] m were selected for the top, middle and bottom substructure, respectively, As is convention, the coordinates x-y, y-z and z-x used here represent the transverse, sagittal and coronal planes, respectively. Second, in order to optimize the rigidity or compliance of the 4B-SPM, the stiffness ellipsoid volume equation $O=\left(4\pi /3\right)k_{a}k_{b}k_{c}\;$was chosen as the objective function to maximize or minimize, here $k_{a}$, $k_{b}$ and $k_{c}$ are the orthogonal axes of the ellipsoid. These two parameters make the problem a bounded nonlinear multi-objective (roll, pitch and yaw axes) optimization problem. Because of the multiple parameters, a genetic algorithm was chosen as the optimization method for determining substructure placement. The genetic algorithm attempts to minimize the objective function, so in order to maximize rigidity and compliance, $O=-\left(4\pi /3\right)k_{a}k_{b}k_{c}$ and $O=\left(4\pi /3\right)k_{a}k_{b}k_{c}$ were used, respectively. For maximizing nonhomogeneous stiffness, the objective function$\;O=-\left(k_{a}-k_{b}-k_{c}\right)$ was used, which drives$\;k_{a}\to \infty$, $k_{b}\to 0$ and $k_{c}\to 0\;$as the objective function is minimized. In this case, maximizing $k_{a}\;$and minimizing $k_{b}$ and $k_{c}$ is the arbitrarily chosen nonhomogeneous behavior. Alternatively, $k_{b}$ or $k_{c}$ could also be maximized if desired.

For executing the genetic algorithm, Matlab’s Optimization Toolbox (Mathworks, MA, USA) was used. The genetic algorithm function (ga) was given the boundary conditions and objective functions stated, along with the stiffness model with shoulder plate orientation as an input and the stiffness ellipsoid as an output. The shoulder plate orientation was varied in 10° along the pitch and yaw Euler angles and bounded by the octant (+x, +y, −z). At each orientation, the genetic algorithm was executed and the optimal substructure mounting points were found. The approach generates a point cloud of best solutions for each substructure mounting location. The mean of these point clouds is taken as the generalized best solution.

In addition to maximum, minimum and nonhomogeneous stiffness models developed for the shoulder, a fourth model is developed for the hip joint. This is done in an effort to demonstrate the versatility of the 4B-SPM architecture and the stiffness analysis used. In this fourth model, the maximum stiffness ellipsoid is determined along with the corresponding mounting point positions. This model was developed in the same manner as the shoulder model. Each mounting point solution was restricted to the following geometric volumes in order to produce a viable solution that interfaces well with the hip [$-0.5<x_{t}<-.2,$ $-0.1<y_{t}<0.2,\;\;$ $0.2<z_{t}<0.4$] m [$-0.2<x_{m}<0.2,$ $-0.3<y_{m}<-0.1,\;$ $0.2<z_{m}<0.4$] m, and [$0.1<x_{b}<0.4,$ $-0.1<y_{b}<0.1,\;$ $0.2<z_{b}<0.4$] m, The workspace was bounded by the following three thigh orientations: 90° flexion, 45° adduction and at rest.

## 3 Results

### Stiffness Model Testing

**FIGURE 7:**
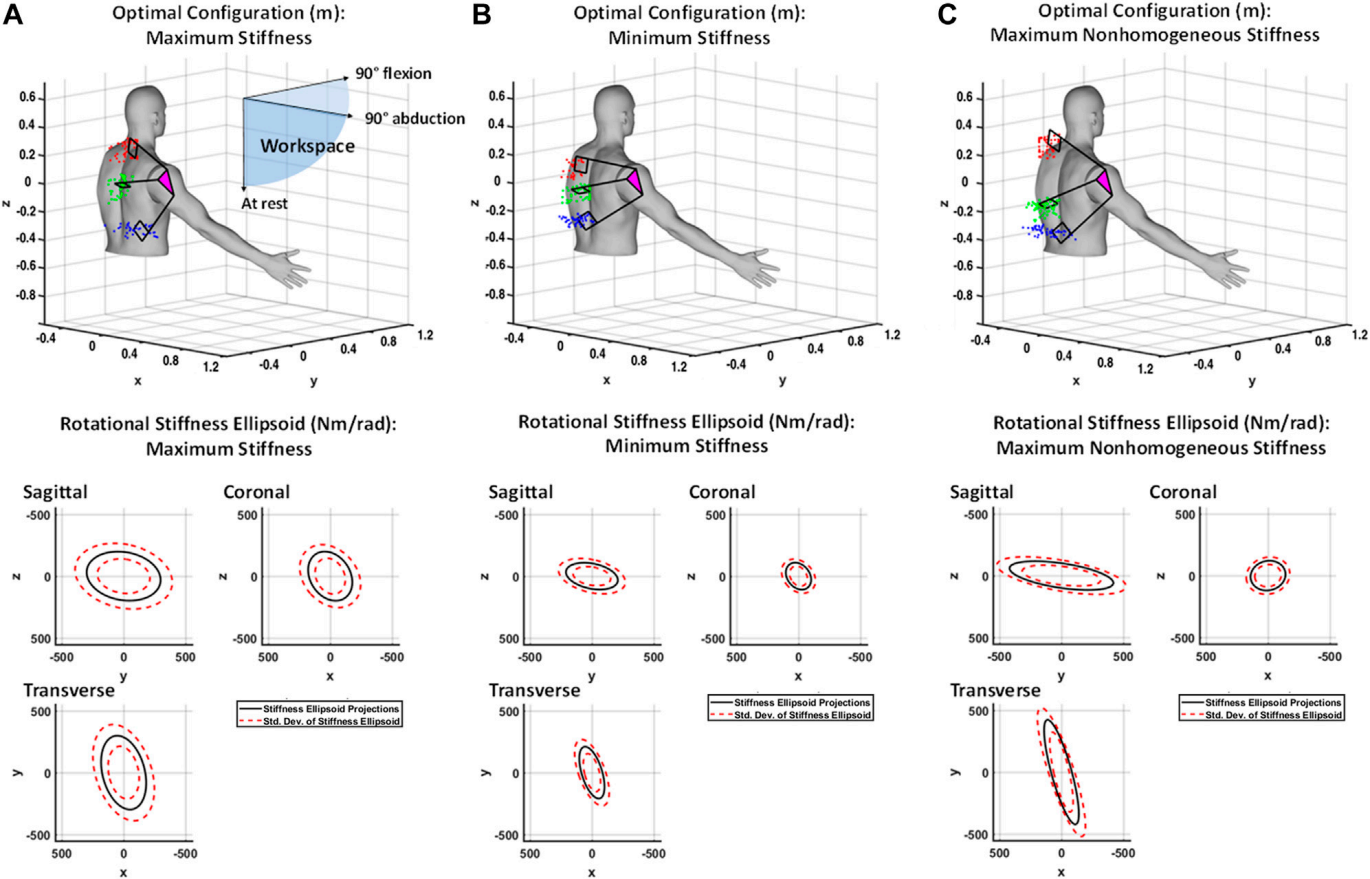
Orientation of the shoulder exoskeleton along with projections of the associated theoretical rotational stiffness ellipsoid (Nm/rad) shown in black. The roll, pitch and yaw stiffness measurements are shown in red for contrast. The origin of the frame is at the center-of-rotation of the human shoulder.

A comparison of the theoretical and mean measured stiffness is shown in Figure 7 for the shoulder plate orientated at 90° flexion. The mean error along roll-pitch-yaw is 11.8% with a standard deviation of 8.4. While error does exist, it should be noted that the size and shape of the theoretical model demonstrates a reasonable approximation of stiffness based on the global axis measurements taken.

Several causes for the error have been identified by the authors: 1) Imperfect intersection of the roll axes for the three substructures. This misalignment could produce increased resistance to applied torque that may contribute to differing stiffness results. This could be corrected with higher manufacturing tolerances. 2) Backlash in the servo motors. This could potentially cause play in the shoulder plate that could affect the stiffness measurements. It should be noted that efforts to minimize backlash were taken by applying minor tension of the three substructures against the shoulder plate equal to the measured backlash of the servos. This minimizes backlash without changing the kinematic solution. 3) Imperfect modeling of the prototype’s geometric and material properties. Measurements taken from the prototype and materials utilized vary within tolerance. These tolerances are not accounted for by the theoretical model and are therefore a potential source for minor error. 4) Simplification 4-bar mechanism nodal diagram, as described in Section 2

### Stiffness Optimization

For the octant workspace bounded by the +x, +y, and −z axes defined in Figure 5, the 4B-SPM substructure configurations to achieve optimal rigid, compliant and nonhomogeneous stiffness behavior were found. The optimal configurations are shown in Figure 5, along with a point cloud of best solutions for different shoulder plate orientations. These solutions were found at 10° increments along the pitch and yaw Euler angles. The optimal substructure configuration for each result is taken to be the mean location of each substructure point cloud. For optimal rigidity, the virtual center of each point cloud for the top, middle and bottom substructure, respectively, are $A_{t}=$ [−0.23, −0.16, 0.27]^T^ m$\;A_{m}=$ [−0.27, −0.21, 0.02]^T^ m and $A_{b}=$[−0.21, −0.12, −0.31]^T^ m. For optimal compliance, the virtual center of each point cloud for the top, middle and bottom substructure, respectively, are $A_{t}=$ [−0.25, −0.16, 0.11]^T^ m$\;A_{m}=$ [−0.29, −0.23, 0.01]^T^ m and $A_{b}=$[−0.28, −0.14, −0.24]^T^ m. For the optimal nonhomogeneous stiffness behavior, the virtual center of each point cloud for the top, middle and bottom substructure, respectively, are $A_{t}=$ [−0.29, −0.24, 0.29]^T^ m $A_{m}=$[−0.29, −0.24, −0.10]^T^ m and $A_{b}=$[−0.21, −0.14, −0.26]^T^ m. The generalized rotational stiffness ellipsoid that represents the average stiffness across the entire workspace for each solution is shown in Figure 5 as well. Included with them is the standard deviation for each solution.

The results shown in Figure 5 help identify a few interesting characteristics of the 4B-SPM. Firstly, a comparison between maximum rigidity and compliance suggests that stiffness is largely dependent on the distances between substructures mounts. This is somewhat intuitive, although the extent of dependency was not clear until now. Another interesting feature identified by these findings is how the rigid and compliant results show fairly symmetric solutions corresponding to relatively homogeneous stiffness ellipsoids. In contrast, the nonhomogeneous stiffness results shown in Figure 5C correspond to a highly nonsymmetrical substructure mounting point solution. These observations would suggest that symmetry of the 4B-SPM affects its degree of homogeneous stiffness behavior.

The results shown in Figure 5 also provide the opportunity to compare the stiffness of this new 4B-SPM architecture to that of the previous motion-coupled SPM architecture developed by the authors for similar purposes and discussed in the Introduction. In prior work the authors analyzed the rotational stiffness of this motion-coupled design across the same workspace used in this paper for the 4B-SPM (Hunt et al., 2018). For a maximum stiffness configuration, the motion-coupled design had a mean stiffness ellipsoid volume of $6.22\cdot 10^{6}{\left(Nm/rad\right)}^{3}$. In comparison, the 4B-SPM has a mean stiffness ellipsoid volume of $3.24\cdot 10^{7}{\left(Nm/rad\right)}^{3}$ for the maximum stiffness configuration. This increase in stiffness is likely due to 1) the addition of the three revolute actuators that control the roll of each 4B-SPM substructure and 2) the simplified 4-bar design that possess fewer failure modes. Other factors, such as part materials and geometry may also contribute to the increased stiffness.

**FIGURE 8:**
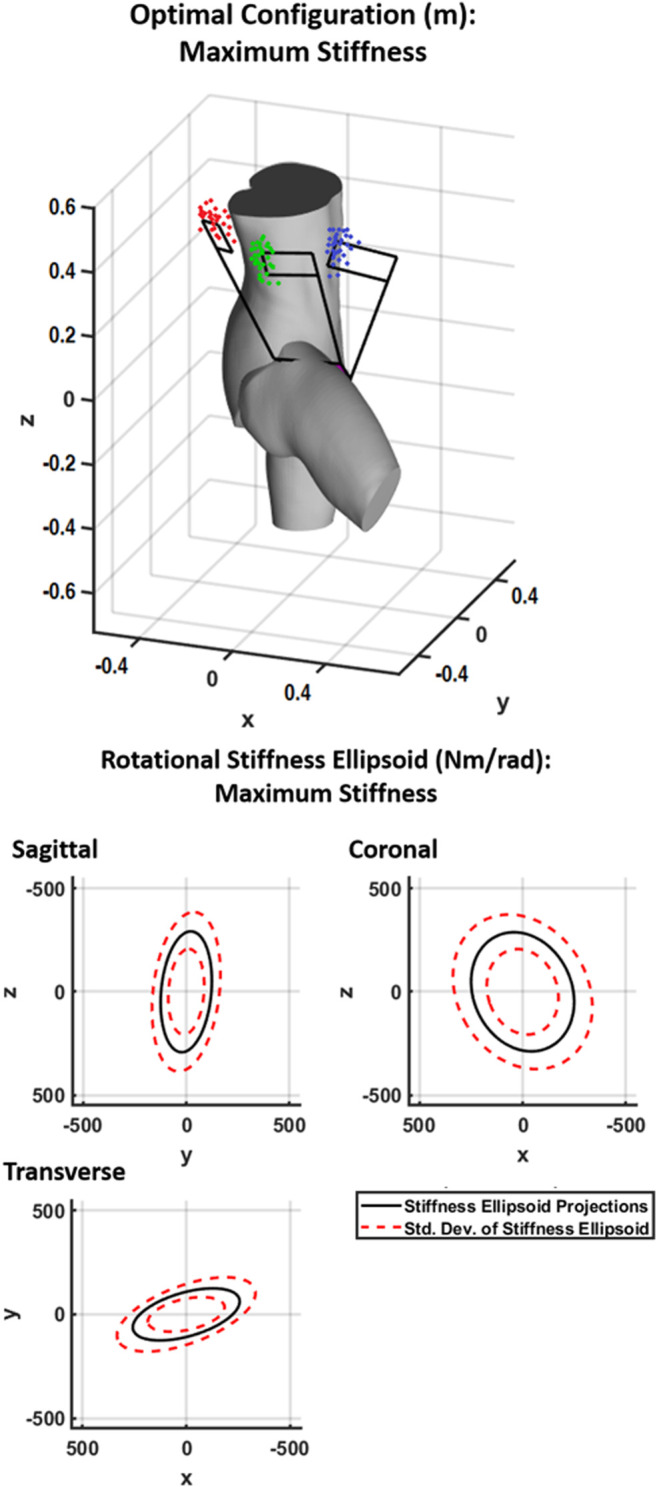
Shown at top is the generalized maximum stiffness configuration for the 4B-SPM hip exoskeleton substructures along with point clouds of the best solutions found throughout the workspace. Shown at bottom are projections of the generalized maximum stiffness ellipsoid.

In addition to the findings presented for the 4B-SPM shoulder exoskeleton, the maximum stiffness results of a theoretical hip exoskeleton are also presented. These results are shown in Figure 8. For optimal rigidity, the virtual center of each point cloud from left (red) to right (blue) are $A_{t}=$ [−0.37, 0.11, 0.38]^T^ m$\;A_{m}=$ [−0.07, −0.18, 0.39]^T^ m and $A_{b}=$[0.22, 0.34, 0.4]^T^ m, respectively. As previously mentioned, this second embodiment of the 4B-SPM architecture is included here in order to demonstrate the versatility of the 4B-SPM architecture and the stiffness analysis used. It should be noted that the choice of a hip exoskeleton was arbitrary. This second embodiment could have just as easily been a 4B-SPM exoskeleton wrist or ankle alternative.

## 4 Discussion

The work performed for this paper was motivated by the need for exoskeleton architectures that are capable of matching the workspace of a user while exhibiting desired stiffness characteristics. Because of limitations in the stiffness or workspace of typical serial and parallel actuated architectures, the authors developed the new 4B-SPM architecture in prior work that was specifically designed for exoskeleton applications involving complex biological joints like the shoulder, hip, wrist and ankle. Demonstrated in the form of a shoulder exoskeleton, the authors performed a dynamic analysis on the 4B-SPM in order to help validate the derived stiffness model. The model was then used to optimize the 4B-SPM configuration in order to achieve rigid, compliant and nonhomogeneous stiffness behavior.

The results of this paper detail a theoretical stiffness model for the 4B-SPM presented, along with an experiment to validate the model. An error between the prototype stiffness and theoretical stiffness of 11.8% with a standard deviation of 8.4 was reported. Despite some error, the model still proved to be a reasonable approximation of stiffness. Possible causes for the error are discussed in Section 3.1

The stiffness model was used in conjunction with a bounded nonlinear multi-objective optimization method in order determine the optimal placement of the three actuated substructures to achieve certain dynamic behavior within a given workspace. The workspace was chosen to be one octant of a sphere defined by the three arm orientations: 90° flexion, 90° abduction, and at rest. For this workspace, the actuator placements for optimal rigid, compliant and certain nonhomogeneous stiffness behavior were demonstrated.

The main contribution of this work is providing researchers and members of the robotics community who chose to use the 4B-SPM architecture a means of adjusting its dynamic performance to fit many different exoskeleton applications. To reiterate, there are many reasons to use the 4B-SPM, the primary ones being: 1) interfaces well the shoulder, hip, wrist and ankle; 2) does not require any complex mechanical components; 3) has very flexible actuator placement; and 4) does not require the human joint for a singular kinematic solution. With the addition of the presented stiffness model, future wearable 4B-SPM devices could be optimized for a variety of tasks and applications, such as lifting, jumping, running, crush protection and impact absorption.

## Data Availability

The raw data supporting the conclusions of this article will be made available by the authors, without undue reservation.
